# Acceptability and Feasibility of Implementing a Telehealth Kit for Older Adults with Diabetes in an FQHC Clinic

**DOI:** 10.21203/rs.3.rs-10155275/v1

**Published:** 2026-07-27

**Authors:** Hongmei Wang, Alauna M. Hunt, Stephanie Merem, Omolola E. Adepoju

**Affiliations:** Texas Southern University; University of Houston; Texas Southern University; University of Houston

## Abstract

**Background:**

Telehealth-supported diabetes monitoring may improve diabetes monitoring for older adults who experience barriers to traditional in-person care, but less is known about the acceptability and feasibility of telehealth-supported diabetes management in Federally Qualified Health Center (FQHC) settings. This pre-post quasi-experimental study examined the acceptability and feasibility of implementing a telehealth kit to support diabetes monitoring among adults aged 50 years and older receiving care at an FQHC clinic.

**Methods:**

This single-group pilot feasibility study enrolled adults aged 50 years and older with diabetes who received a telehealth kit that included a Bluetooth-enabled glucometer, blood pressure monitor, testing supplies, written instructions, and in-person training. No randomization or comparator group was used, and the study was not designed to test the efficacy of a new diabetes treatment. Quantitative surveys and glucose data were collected at baseline, 30 days, and 60 days to describe feasibility indicators and exploratory glucose trends. Qualitative open-ended responses were collected at baseline and follow-up visits and analyzed using rapid thematic analysis to identify barriers and facilitators to telehealth engagement.

**Results:**

Thirty participants completed the study. Participants were predominantly female (60.0%) and Non-Hispanic Black (66.7%); all reported smartphone and internet access, and most reported medium-high or high digital confidence. Mean glucose values remained relatively stable from baseline to 30 and 60 days, with overall means of 122.7, 127.9, and 117.4, respectively. Participants younger than 65 years experienced a significant reduction in glucose at 60 days compared with those aged 65 years and older. No significant differences in glucose change were observed by education, digital confidence, or medication adherence. Qualitative findings identified four themes: acceptability and usefulness of the telehealth platform over time; platform usability and authentication barriers; the role of digital literacy and support systems in participation; and contextual and infrastructural barriers beyond platform usability.

**Conclusions:**

Telehealth-supported diabetes monitoring was acceptable and feasible for some older adults receiving care in an FQHC setting, but engagement was conditional on reliable connectivity, usable platform design, successful authentication, and ongoing support. Findings suggest that future feasibility and implementation in medically underserved settings should incorporate simplified technology workflows, structured onboarding, technical assistance, and, when appropriate, family or caregiver support.

## Introduction

Type 2 diabetes is a major and growing public health concern in the United States, with prevalence rates increasing substantially among older adults. Approximately 29% of adults aged 65 years and older have diabetes, and diabetes in later life is associated with increased morbidity, mortality, healthcare utilization, and poor quality of life.^1,2^ The burden of diabetes is especially high among vulnerable older adults served by Federally Qualified Health Centers (FQHCs), which provide comprehensive primary care to medical underserved communities regardless of ability to pay.^3,4^ FQHC patients often face multiple social and behavioral challenges, such as poverty, limited health literacy, transportation barriers, food insecurity, and lack of access to specialty care.^5^ These challenges complicate diabetes care, as effective management requires sustained engagement in complex self-care behaviors, including blood glucose monitoring, medication adherence, dietary modification, physical activity, and ongoing access to healthcare providers.^6^ These demands can be difficult for many older adults, particularly those served in FQHCs. In FQHC settings, diabetes management is not only a matter of individual behavior but also reflects broader challenges in healthcare access, technology access, community resources, and continuity of care.

Traditional models of diabetes care rely heavily on frequent in-person clinic visits for monitoring, education, and medication adjustment. However, older adults served by FQHCs frequently encounter substantial barriers to attending regular appointments. Telehealth and mobile health may offer promising ways to support diabetes care by expanding access to monitoring, education, and clinical feedback outside traditional clinic settings.^7-10^ Telehealth approaches can include remote monitoring of glucose and blood pressure, diabetes education, medication review, and timely communication with care teams.^11^ Prior studies suggest that these approaches may improve glycemic control, self-management confidence, and patient-provider communication.^7-9^ These approaches may be especially useful for patients who face transportation difficulties, limited clinic availability, mobility limitations, or competing financial and caregiving responsibilities.^12,13^ However, the existing evidence also indicates that the outcomes are not uniform across populations or settings. Outcomes may vary depending on program design, intensity of support, patient digital literacy, broadband or device access, health literacy, caregiver involvement, and integration into clinical workflows.^14-16^ Although prior research has demonstrated the potential benefits of telehealth for diabetes management, less is known about the acceptability and feasibility of these approaches for older adults receiving care in FQHCs. Therefore, more research is needed to understand not only whether telehealth can improve diabetes-related outcomes, but also how older adults in medically underserved communities experience, adopt, and sustain telehealth use in real-world primary care settings.

This pilot mixed-methods pre-post quasi-experimental study explored the acceptability and feasibility of implementing a telehealth kit to support diabetes monitoring among older adults receiving care at an FQHC. Specifically, the study examined whether older adults perceived the telehealth kit as usable, acceptable, and supportive of diabetes self-management, and identified barriers and facilitators that shaped their engagement with it. By focusing on implementation-relevant outcomes, this study contributes to the literature by highlighting factors that may influence the successful adoption, sustainability, and scalability of telehealth support for diabetes monitoring in safety-net FQHC settings.

## Methods

### Study Design

**Figure 1:**
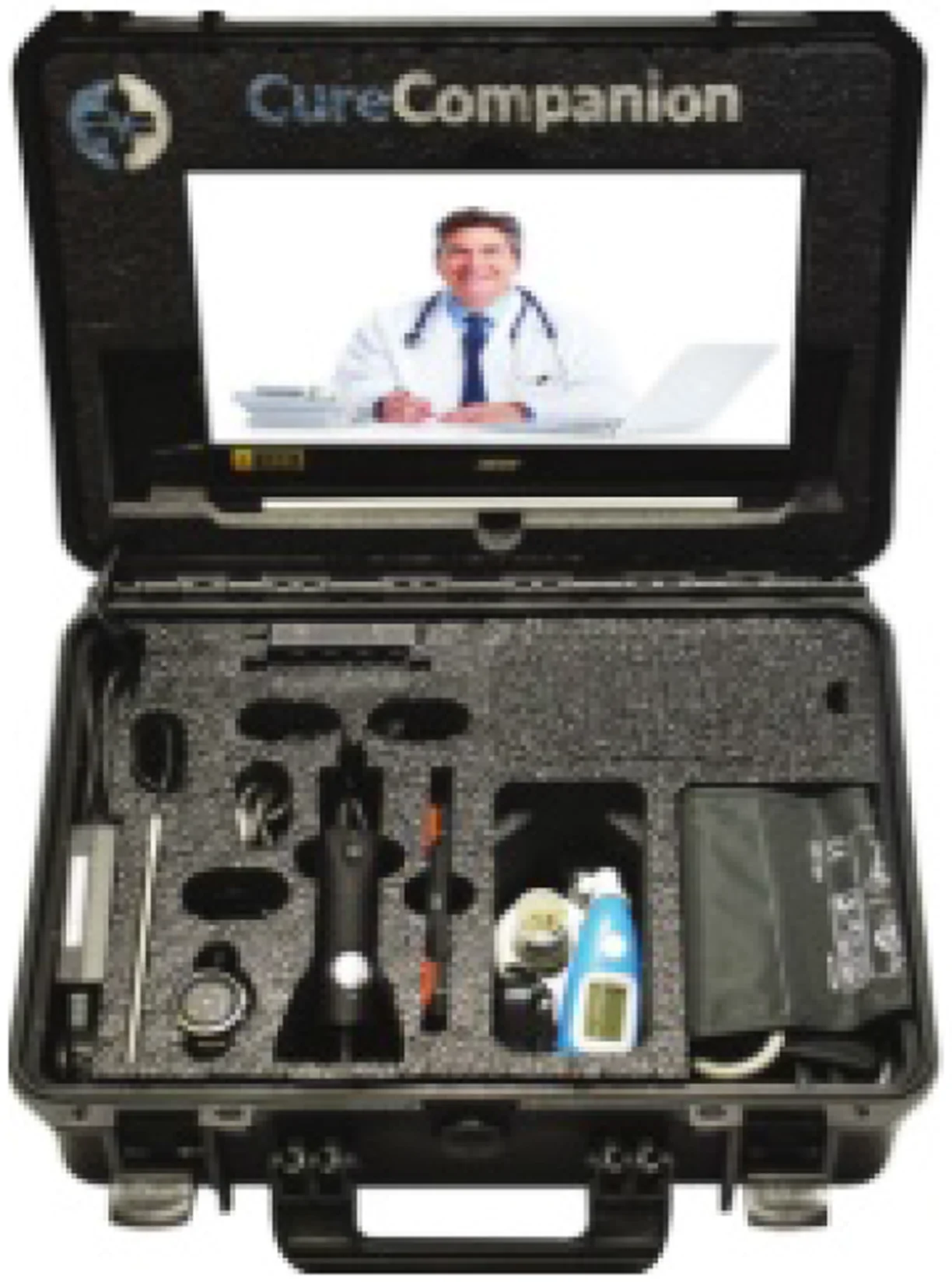
Telehealth Kit

This pilot study used a single-group, mixed-methods, pre-post quasi-experimental design to examine the acceptability and feasibility of using a telehealth kit for diabetes monitoring among adults aged 50 years and older receiving care at a FQHC clinic. No randomization, allocation, or comparator group was used. The study was designed to evaluate implementation-relevant outcomes, including usability, acceptability, engagement, barriers, facilitators, and exploratory glucose trends; it was not designed to test the efficacy of a new diabetes treatment. Enrolled study participants received a telehealth glucose monitoring kit, a 13-inch, 6.5-pound rectangular box that included a Bluetooth-enabled glucometer, blood pressure monitor, test strips, lancets, and written instructions for device setup and use. [Figure 1]. A trained research coordinator conducted one-on-one, in-person training at enrollment to orient participants to the kit, provide hands-on guidance on device setup, and ensure participants could successfully collect and transmit data from the glucometer and blood pressure monitor.

#### Setting

The study was conducted at an academic, community-based primary care clinic affiliated with a School of Medicine. The clinic operates as an FQHC look-alike and is located in a medically underserved area of Houston. It serves a diverse patient population, including individuals with low incomes, uninsured and underinsured patients, older adults, and patients from a variety of racial and ethnic backgrounds. The clinic provides comprehensive primary care services and incorporates behavioral health, preventive care, chronic disease management, and connections to community resources as part of its care model.

#### Participants

Eligible participants were adults aged 50 years and older with a documented diagnosis of diabetes who received care at the partnering FQHC Look-Alike clinic. An additional inclusion criterion was the ability to speak and understand English to ensure comprehension of study procedures and survey materials. Participants were excluded if they met any of the following criteria: (1) Documented alcohol or substance use disorder within the three months preceding enrollment, (2) Active cancer diagnosis at the time of screening; (3) Current enrollment in another research study with potential to affect diabetes monitoring or study outcomes; (4) Cognitive impairment or other medical/psychiatric conditions that interfered with the ability to provide informed consent.

A total of 34 individuals were enrolled, of whom 30 completed all phases of the study. Four participants were lost to follow-up after we attempted to reach them 3 or more times during subsequent survey waves. Each participant received $50 grocery card in compensation for their involvement at each time point.

#### Data Collection and Measures

Data were collected at three time points: baseline, first follow-up (30 days), and second follow-up (60 days). At baseline, participants completed structured surveys and open-ended questions to assess demographic characteristics, access to technology, and experiences with diabetes self-management. Glucose values were summarized at baseline, 30 days, and 60 days to describe trends during the feasibility period. At the 30- and 60-day follow-up visits, data collection consisted solely of questions focused on participants’ experiences with the telehealth kit, including perceived benefits, barriers, and facilitators of use. These questions assessed how participants engaged with the technology over time and explored factors influencing sustained adoption and usability.

Quantitative survey items assessed demographic variables (e.g., age, sex, race/ethnicity) and technology access. Technology-related measures included device ownership, internet use, comfort with digital tools, and perceived challenges with technology-supported diabetes care. Qualitative questions were designed to elicit participants’ experiences with technology, barriers, and facilitators related to self-management using the telehealth kit, and overall impressions of the telehealth kit. Responses were captured verbatim.

### Data Analysis

Descriptive statistics were used to summarize the study sample, including technology access, digital confidence, medication adherence, and glucose trends. Given the pilot feasibility focus and absence of randomization or a comparison group, quantitative analyses were interpreted as exploratory and hypothesis-generating rather than as definitive tests of clinical efficacy. Qualitative methods, including rapid thematic analysis, were used to identify barriers and facilitators to using the telehealth kit. The study was approved by the University of Houston Institutional Review Board (IRB #: STUDY00004370). All participants provided written informed consent prior to enrollment. Data were de-identified and stored in secure, access-controlled systems that comply with institutional data protection requirements.

Qualitative analysis focused on identifying common patterns related to technology use, barriers and facilitators to diabetes self-management, and participants’ overall impressions of the telehealth kit. The responses were reviewed multiple times to gain familiarity with the data. During this initial review, notes were made on repeated ideas, meaningful phrases, and early impressions regarding telehealth kit use. Next, an initial coding process was conducted. Codes were assigned to segments of text describing participants’ experiences with the kit's acceptability, challenges, perceived benefits, support needs, and confidence in digital literacy. Themes were then developed from these categories to represent the major findings across participant responses. Potential themes included confidence in technology and usability, barriers to telehealth kit use, and overall acceptability of the kit. Themes were reviewed and refined to accurately reflect the data and address the study’s purpose.

## Results

Table 1 displays the baseline characteristics of study participants. A total of 30 participants completed the study. The sample was predominantly female (60%), while 40% were male. The majority of participants identified as Non-Hispanic Black (66.7%), followed by Hispanic (20.0%), and other races (13.3%).

Educational attainment varied across the sample. Approximately 47% had a high school degree or less, while 53.3% held more than a high school degree.

Participants generally reported strong digital competency. Half (50%) rated their digital confidence as high, and 33.3% reported medium-high confidence. Only 10.0% rated their digital confidence as medium-low, while 6.7% rated themselves as low. All participants reported using a smartphone and having access to the internet. The most common form of home internet access was Wi-Fi connection (36.7%), followed by cellular service (33.3%), then broadband (30.0%). In response to prompts about self-rated general health, most of participants reported very good health (40%), or good health (40%) followed by fair health (16.7%), while 3.3% rated their health as excellent. With regards to medication non-adherence, most of the participants reported perfect medication adherence (56.7%), followed by moderate non-adherence (33.3%), high non-adherence (6.7%), while only 3.3% reported mild non-adherence (3.3%).

Table 2 depicts mean glucose levels at baseline and at follow-up by selected patient characteristics. Glucose levels remained relatively stable over the feasibility period with mean values of 123 at baseline, 128 at 30 days, and 119 at 60 days. Exploratory analysis was used to assess changes in glucose levels over time (baseline, follow-up 1, follow-up 2) and to evaluate whether these changes differed across subgroups. Compared with participants aged 65 + years, participants younger than 65 years had significant reductions in blood glucose at follow-up 2 (p = 0.04), but not at follow-up 1 (p = 0.16). No significant differences in glucose changes over time were observed across education, digital confidence, or medication adherence groups (all p > 0.05).

Open-ended responses were analyzed at baseline, 30 days, and 60 days to understand participants’ experiences with the telehealth platform for diabetes monitoring. All 28 participants completed the baseline open-ended question; 18 participants responded at 30 days, and 16 responded at 60 days. Table 3 showed four primary themes that emerged: 1. Acceptability and usefulness of the telehealth platform over time; 2. Platform usability and authentication barriers; 3. Digital literacy and support systems shaped participation; 4. Contextual and infrastructural barriers beyond platform usability.

### Theme 1: Acceptability and usefulness of the telehealth platform over time

Overall, many participants described the telehealth platform as acceptable and manageable, particularly when they did not encounter technical problems. At baseline, several participants reported minimal or no barriers and described the tool positively: “No barriers in particular to note” (P3) and “it’s great” (P2). This pattern continued at follow-up for some participants, who reported that uploading values and accessing the platform were straightforward. One participant stated, “I haven’t had any trouble uploading my values; I had reliable internet access” (P4a). Another reported, “I did not have any trouble with the tablet, no interruption in service…” (P25a). By 60 days, some participants continued to report smooth use of the tablet, CureCompanion app, and internet access, including one participant who “has not experienced any barriers when using the tablet, CureCompanion app, or having internet access” (P2b).

Participants also described the platform as convenient and helpful for diabetes self-management. At 30 days, one participant noted, “The tablet has made keeping track of his levels and managing his diabetes more convenient” (P2a). At 60 days, another participant “felt that this platform was useful in monitoring A1C levels…” (P10b). These responses suggest that when technical access was reliable and the platform functioned as intended, participants perceived the telehealth tool as a useful adjunct to diabetes monitoring.

### Theme 2: Platform usability and authentication barriers

Despite overall acceptability among many participants, usability problems emerged as a recurring barrier over time. Authentication issues, including remembering passwords, resetting passwords, and being locked out of the app, were commonly described. At 30 days, one participant identified the main issue as “just remembering their password” (P1a), and another participant reported, “It wouldn’t recognize my ‘code’… having to reset it several times” (P28a). By 60 days, these problems persisted for some participants. One stated, “I did frequently forget my password and had to reset it…” (P6b), while another reported, “I locked myself out of the [CureCompanion] app and couldn’t upload values…” (P28b).

Participants also reported difficulties with platform navigation and app-related glitches. At 60 days, participants described broader usability challenges, including difficulty navigating the Android tablet interface and app instability. One participant stated, “I had issues navigating the Android platform of the tablet” (P11b). Another noted, “The app was not user-friendly as it would often ‘crash’…” (P23b). These issues interfered with participants’ ability to independently upload values and, in some cases, required alternative methods of reporting.

Some participants developed workarounds to remain engaged in the study despite platform barriers. For example, one participant reported, “Had difficulty reporting my values on the tablet, so used my laptop instead” (P11b). Another stated, “Was unable to log my values, so I had to send them to [research coordinator] to upload” (P23b). These workarounds suggest that participants were motivated to continue monitoring but required flexible pathways when the telehealth platform was difficult to use.

### Theme 3: Digital literacy and support systems shaped participation

Participants’ ability to use the telehealth tool was shaped by digital literacy, prior experience with technology, and access to support. Some participants attributed barriers to limited technology knowledge or user error. At 30 days, one participant stated, “Any barriers… were due to user error and lack of tech knowledge” (P11a). At 60 days, another participant described broader digital access and skill challenges: “I don’t know how to properly use the digital devices… Right now, I do not have reliable cellphone or internet services…” (P12b).

Family assistance was an important facilitator of engagement for some participants. At 30 days, participants described receiving help from family members to learn the equipment or complete monitoring tasks. One stated, “My grandson was able to teach me how to properly use the equipment…” (P15a). Another noted, “My daughter has to help me take my A1C and [BP]” (P9a). These responses indicate that family members helped reduce the burden of technology use and supported continued participation.

Participants also offered concrete recommendations to reduce the technology burden and improve usability. Suggestions included additional education, alternative authentication options, and more embedded guidance within the tablet. One participant stated, “Educating your clients would be more beneficial…” (P26a). Another suggested, “facial recognition instead of having to remember a password…” (P26a). Participants also recommended adding practical resources to the device, such as an “update on the tablet [with] helpful tips on where we can go to get care…” (P26b). These recommendations reflect participants’ desire for a more supportive, intuitive, and accessible telehealth experience.

### Theme 4: Contextual and infrastructural barriers beyond platform usability

Participants’ engagement with the telehealth platform was also shaped by contextual and infrastructural barriers beyond the platform itself. Connectivity was a recurring example. At 30 days, one participant reported “frequent loss of internet connection… prevented me from logging in” (P6a). At 60 days, another noted that it was “hard for me to connect to Wi-Fi once I left my house” (P19b). These responses suggest that internet access was neither fixed nor universally available; even participants who could connect in one setting encountered barriers when moving outside their usual environment. Thus, connectivity functioned less as a platform usability issue and more as an access-related condition that shaped whether participants could consistently engage with telehealth.

Physical limitations and the burden of supplies also affected participation. One participant reported that “lack of mobility issues prevents me from using equipment” (P9a). Another described the time and supply burden associated with glucose monitoring: “The glucometer takes a lot of time… I have to do it 2 or 3 times… I run out of supplies quickly” (P27a). At follow-up, participants suggested that additional materials, including “more materials (lancets)” (P15a), would help support continued use.

Other barriers included language, trust and security concerns, life circumstances, and preferences for in-person care. One participant described concerns from a family member, stating, “My sister would warn against AI and Tech in medicine…” (P13a). At 60 days, another participant reported, “There was also a language barrier” (P6b). Life circumstances also disrupted consistent engagement, as reflected by one participant who was “unable to upload consistently due to the extenuating circumstances I’ve been having at home” (P13b). Finally, some participants continued to value in-person care and social connection. One participant stated, “I prefer… going physically is also an opportunity to meet people so I prefer to go into the doctor’s office…” (P27a). These responses demonstrate that telehealth engagement was shaped not only by platform design, but also by participants’ social context, physical capacity, home environment, and care preferences.

## Discussion

This pilot mixed-methods pre-post quasi-experimental study examined the acceptability and feasibility of implementing a telehealth kit to support diabetes monitoring among older adults receiving care at an FQHC. Quantitative findings showed that participants entered the study with high digital access, as all participants reported smartphone use and internet access, and most reported medium-high or high digital confidence at baseline. Mean glucose values remained relatively stable across baseline, 30 days, and 60 days, with values of 123, 128, and 119, respectively. Although the glucose change was limited, exploratory subgroup analysis showed a significant reduction in glucose at 60 days among participants younger than 65 years compared with those aged 65 years or older. No significant differences in glucose change were observed by education level, digital confidence, or medication adherence. Qualitative findings helped explain these quantitative patterns. Open-ended responses collected at baseline, 30 days, and 60 days showed that although the platform was acceptable and useful for some participants, engagement was shaped by four primary themes: acceptability and usefulness of the telehealth platform over time; platform usability and authentication barriers; digital literacy and support systems shaping participation; and contextual and infrastructural barriers beyond platform usability. These findings suggest that telehealth-supported diabetes monitoring was feasible and potentially useful, but engagement was shaped by individualized factors, including age, platform usability, digital literacy, support availability, connectivity, and broader life circumstances. Because this was a single-group pilot feasibility study, glucose findings should be interpreted as exploratory rather than evidence of clinical efficacy.

The relative stability of glucose values across the study period suggests that the telehealth platform may have supported maintenance of glycemic status rather than producing substantial overall improvement. This finding remains clinically relevant because maintaining stable glucose levels may be meaningful for individuals with diabetes who face social, technological, and health-related constraints. The finding is consistent with previous studies showing that telehealth can sustain, but not always markedly improve glycemia.^17-19^ However, the significant reduction in glucose among participants younger than 65 suggests that age may influence the extent to which individuals benefit from telehealth-supported monitoring. A study showed that HbA1c significantly reduces primarily among adults aged 40–65 years, while improvements plateau after age 65 and among older adults. This may reflect age-related differences in comfort with digital tools, in the ability to troubleshoot technical problems, and in familiarity with app-based workflows. Given the pilot pre-post design, these age-related findings should be viewed as preliminary signals to inform adequately powered future studies rather than as confirmatory evidence of effectiveness.

Although participants generally reported strong digital confidence at baseline, the follow-up results showed a variety of digital and technical challenges. Education level also did not significantly differentiate glucose change. This suggests that formal education alone may not determine telehealth benefit or engagement. The findings suggest that self-rated confidence or education level may not fully capture the specific practical skills required to consistently use a telehealth platform. Participants may feel comfortable using smartphones or the internet but still struggle with platform-specific demands, including password management, authentication codes, app lockouts, tablet navigation, app crashes, or glucose upload procedures. Early work found that adults with type 2 diabetes could report confidence in using mobile eHealth technologies, yet relatively few used health apps in daily life.^20^ Similarly, a study emphasized that digital health engagement requires specific competencies, and many older adults required support to complete these tasks.^21^ Therefore, digital confidence should not be treated as equivalent to telehealth readiness. Future telehealth interventions should effectively engage in digital health communication and management by integrating patient education and family support.

Medication adherence also did not significantly modify glucose change over time, despite high baseline adherence. This may be explained by limited variability in adherence, the short follow-up period, or the influence of other self-management factors beyond medication-taking. Glucose values may also have been affected by many non-pharmacologic-related factors, such as diet, physical activity, glucose monitoring, socioeconomic factors, and health system engagement.^22,23^ Our study qualitative responses supported barriers influencing diabetes management, such as mobility limitations, running out of lancets, language barriers, concerns about technology, and inconsistent internet access. These findings reinforce that medication adherence for diabetes control occurs within a complex self-management environment.

A central qualitative finding was that participants viewed the telehealth platform as acceptable and useful when it functioned reliably. Some participants reported no major barriers, reliable internet access, and ease of uploading glucose values. Others described the platform as making diabetes monitoring more convenient. However, acceptability was conditional. Positive experiences depended on reliable connectivity, successful authentication, and the absence of repeated technical problems. Usability and authentication barriers were among the most direct obstacles to engagement. Participants described difficulty remembering passwords, resetting codes, being locked out of the application, navigating the tablet interface, and experiencing app crashes. These issues prevented independent uploading and reduced engagement. These findings are consistent with previous studies showing that patients found telehealth acceptable and useful when platforms worked reliably and consistently.^24-27^ Technical failures (including unstable connections and failed data transmissions) can reduce satisfaction and sometimes necessitate in-person care.^28^ These findings are also consistent with prior qualitative research on barriers to diabetes education and care. Engagement with diabetes self-management education was shaped by perceived need and practical barriers, including scheduling and transportation challenges.^29,30^ Although the present study focused on telehealth rather than in-person education, a similar pattern was observed: participants’ engagement was shaped by both perceived usefulness and practical access barriers. Telehealth may reduce some barriers associated with transportation and scheduling, but it can introduce new barriers related to authentication, connectivity, device navigation, and digital literacy.

In general, this study has several implications for the implementation of telehealth kits in FQHC and other medically underserved settings. First, telehealth programs should include structured onboarding and hands-on practice with device setup, login, data transmission, and troubleshooting. Second, programs should provide ongoing technical support rather than assuming that baseline digital confidence is sufficient. Third, telehealth platforms should be designed with simplified authentication processes, accessible navigation, and clear instructions. Finally, implementation should include personalized support and, when appropriate, family or caregiver involvement.

### Strengths and limitations

This study has several strengths. The mixed-methods pre-post design enabled the interpretation of exploratory glucose trends alongside participants' lived experience of platform use. The collection of open-ended responses at baseline, 30 days, and 60 days also provided insight into how experiences evolved over time. In addition, the study examined digital confidence, family support, and age as factors that may influence engagement with telehealth-supported diabetes monitoring, enabling a more nuanced interpretation of feasibility and acceptability in an FQHC setting.

This study also has limitations. The single-group design, lack of randomization, absence of a comparator group, small sample size, and 60-day follow-up period limit the ability to draw conclusions about clinical efficacy or causality. Response rates to open-ended questions decreased over time, from 28 participants at baseline to 18 at 30 days and 16 at 60 days, potentially limiting the range of qualitative experiences captured later. Participants also entered the study with high levels of reported digital access, which may limit transferability to populations with lower access to smartphones and internet service. Finally, self-reported digital confidence and medication adherence may not fully reflect actual platform-use skills or day-to-day self-management behavior.

## Conclusion

In conclusion, this pilot pre-post quasi-experimental study found that telehealth-supported diabetes monitoring was acceptable and feasible for some participants, but observed glucose changes were modest over 60 days and should be interpreted as exploratory. Quantitative findings showed stable glucose values overall, with greater improvement among participants younger than 65 years. Qualitative findings explained why high levels of digital access and confidence did not eliminate barriers: participants continued to experience platform-specific usability problems requiring ongoing technical support. These findings suggest that telehealth implementation should be understood through a socio-technical and implementation-focused lens, in which feasibility, acceptability, usability, and technical support are central to successful telehealth-supported diabetes monitoring.

## Figures and Tables

**Table 1:** Baseline Characteristics of Study Participants

Characteristic	Category	N	%
Gender	Male	12	40.0
	Female	18	60.0
Age	50–64	12	40.0
	65+	18	60.0
Race/Ethnicity	Hispanic	6	20.0
	Non-Hispanic Black	20	66.7
	Other	4	13.3
Education	High school or less	14	46.7
	More than high school	16	53.3
Digital Confidence	High	15	50.0
	Medium-high	10	33.3
	Medium-low	3	10.0
	Low	2	6.7
Smartphone Access	Yes	30	100
	No	0	0.0
Internet Access	Yes	30	100.0
	No	0	0.0
Internet Type	Broadband	9	30.0
	Cellular	10	33.3
	Wi-Fi	9	36.7
DOSE-NA Category	Perfect adherence (0)	17	56.7
	Mild nonadherence (1–2)	1	3.3
	Moderate nonadherence (3–5)	10	33.3
	High nonadherence (≥ 6)	2	6.7
Self-rated Health	Excellent	1	3.3
	Very good	12	40.0
	Good	12	40.0
	Fair	5	16.7

**Table 2:** Mean Glucose Levels at Baseline and Follow-up by Select Baseline Variables

Overall blood glucose	Glucose Baseline Mean(SD)	Glucose Follow-up 1 (F1) Mean(SD)	Glucose Follow-up 2 (F2) Mean(SD)	p-value Baseline → F1	p-value Baseline → F2
	**122.7 (22.3)**	**127.9 (28.0)**	**117.4 (35.3)**	**0.15**	**0.65**
**Age Group**				0.16	0.04
< 65	133.4 (28.4)	125.2 (19.5)	104.4 (43.0)		
65+	114.9 (13.0)	129.7 (33.2)	129.1 (23.4)		
**Education**				0.49	0.57
High school or less	121.3 (19.1)	128.2 (34.7)	117.5 (15.6)		
More than high school	124.3 (26.4)	127.6 (18.7)	117.3 (54.3)		
**Digital Confidence**				0.32	0.15
Medium high/High	122 (23.5)	130.2 (29.46)	115.66 (36.8)		
Medium Low/Low	128.9 (5.5)	114.7 (13.64)	130.8 (23.8)		
**Medication Adherence**				0.58	0.84
Perfect Adherence/Mild Non-Adherence	120.8 (24.0)	121.7 (17.2)	112.2 (43.6)		
Moderate to High non-Adherence	125.4 (20.9)	137.2 (38.6)	124.9 (18.8)		

**Table 3:** Summary of Qualitative Data Analysis

Theme	Subthemes	Summary of Finding	Representative Quotes
1. Acceptability and usefulness of the telehealth platform over time	1a. Minimal or no technology barriers 1b. Telehealth perceived as convenient and helpful	Many participants reported few or no barriers to using the tablet, app, or internet. Some described the platform as convenient and useful for tracking diabetes-related values over time.	*“No barriers in particular to note” (P3)* *“I haven’t had any trouble uploading my values, I had reliable internet access.” (P4a)* *“The tablet has made keeping tracking of his levels and managing his diabetes more convenient.” (P2a)*
2. Platform usability and authentication barriers	2a. Password resets / being locked out 2b. Platform/app navigation issues 2c. Workarounds to keep participating	Several participants experienced problems with login, passwords, app navigation, and the platform. Some were unable to upload values directly and used alternative methods, such as a laptop or sending values to the research coordinator.	*“Except just remembering their password.” (P1a)* *“I locked myself out of the [CureCompanion] app and couldn’t upload values…” (P28b)* *“The app was not user friendly as it would often ‘crash’…” (P23b)* *“Had difficulty reporting my values on the tablet, so used my laptop instead.” (P11b)*
3. Digital literacy and support systems shaped participation	3a. User error / limited tech knowledge 3b. Family assistance enabled engagement 3c. Participants suggested concrete improvements/training	Digital literacy affected participants’ ability to use the telehealth platform. Family members helped some participants use equipment and complete monitoring tasks. Participants recommended more education, easier login options, and practical guidance within the tablet.	*“Any barriers… was due to user error and lack of tech knowledge.” (P11a)* *“My grandson was able to teach me how to properly use the equipment…” (P15a)* *“My daughter has to help me take my A1C and [BP].” (P9a)* *“Facial recognition instead of having to remember a password…” (P26a)*
4. Contextual and infrastructural barriers beyond platform usability	4a. Connectivity instability and context-dependent access 4b. Physical limitations and device/supply burden 4c. Language, trust/security concerns, and life circumstances 4d. Preference for in-person care and social connection	Barriers were not limited to the telehealth platform. Participants described unstable internet access, mobility limitations, supply shortages, language barriers, concerns about technology, life stressors, and continued preference for in-person visits.	*“Frequent loss of internet connection… prevented me from logging in.” (P6a) “Hard for me to connect to Wi-Fi once I left my house.” (P19b)* *“Lack of mobility issues prevents me from using equipment.” (P9a);* *“The glucometer takes a lot of time… I have to do it 2 or 3 times… I run out of supplies quickly.” (P27a)* *“My sister would warn against AI and Tech in medicine…” (P13a)* *“There was also a language barrier.” (P6b)* *“I prefer… going physically is also an opportunity to meet people…” (P27a)*

## Data Availability

The datasets generated and/or analyzed during the current study are not publicly available due to participant privacy and confidentiality considerations, but are available from the corresponding author on reasonable request and subject to institutional data-sharing requirements.

## References

[1] Centers for Disease Control and Prevention (2026) Explore National, State and County diabetes surveillance data. https://usdss.cdc.gov/diabetes/report.html

[2] Cowie CC, Casagrande SS, Menke A Diabetes in America. 3rd edition. National Institute of Diabetes and Digestive and Kidney Diseases (US). Published online 2018:35.1–35.16 33651524

[3] What is a Federally Qualified Health Center (FQHC)? — FQHC Associates. https://www.fqhc.org/what-is-an-fqhc

[4] O’Brien MJ, Bailey SC, Gregory DL (2023) Screening for Prediabetes and Diabetes in a National Network of Federally Qualified Health Centers: An Observational Study. J Gen Intern Med 38:3541–3548. 10.1007/s11606-023-08402-1 37731136 PMC10713898

[5] Nguyen KH, Giron NC, Cole MB (2024) National Prevalence of Social Risk Factors at Federally Qualified Health Centers. JAMA Intern Med American Med Association 184:980–982. 10.1001/jamainternmed.2024.1881 PMC11184494 38884949

[6] Walker RJ, Strom Williams J, Egede LE (2016) Influence of Race, Ethnicity and Social Determinants of Health on Diabetes Outcomes. Am J Med Sci 351:366–373. 10.1016/j.amjms.2016.01.008 27079342 PMC4834895

[7] Sharma V, Feldman M, Sharma R (2024) Telehealth Technologies in Diabetes Self-management and Education. J Diabetes Sci Technol SAGE Publications Inc 18:148–158. 10.1177/19322968221093078 35485769 PMC10899831

[8] Kramer ES, VanWyk J, Holmstrom H (2022) Telehealth and Diabetes Management. Primary Care - Clinics in Office Practice. W B Saunders 49:631–639. 10.1016/j.pop.2022.04.007 36357067

[9] Bhattacharya B, Kar K, Venkatesh MP, Singh I Technology-driven diabetes management: a review of digital therapeutics, mobile apps, and clinical evidence. Inform Health Soc Care Published online January 2026:1–21. 10.1080/17538157.2025.2609747 41494047

[10] Suratham S, Klainin-Yobas P (2025) The Effectiveness of Telehealth on Glycemic Stability and Quality of Life Among Patients With Type 1 and Type 2 Diabetes: A Systematic Review and Meta-Analysis. Science of Diabetes Self-Management and Care. SAGE Publications Inc 51:674–698. 10.1177/26350106251378717 41266964

[11] Telehealth technology for diabetes care https://telehealth.hhs.gov/providers/best-practice-guides/telehealth-diabetes-care/telehealth-technology-diabetes-care

[12] Heitkemper EM, Mamykina L, Travers J, Smaldone A (2017) Do health information technology self-management interventions improve glycemic control in medically underserved adults with diabetes? A systematic review and meta-analysis. Journal of the American Medical Informatics Association. Oxford University Press. ;24. 10.1093/jamia/ocx025 PMC6080842 28379397

[13] Mayberry LS, Lyles CR, Oldenburg B, Osborn CY, Parks M, Peek ME (2019) mHealth Interventions for Disadvantaged and Vulnerable People with Type 2 Diabetes. Curr Diab Rep 19. 10.1007/s11892-019-1280-9 PMC7232776 31768662

[14] Ahn J, Yang Y, Kim JY, Pahn J, Jang Y (2025) Effectiveness of mHealth-Based Self-Management Interventions on Self-Efficacy in Patients With Type 2 Diabetes: A Systematic Review and Meta-Analysis. Science of Diabetes Self-Management and Care. SAGE Publications Inc 51:517–531. 10.1177/26350106251361360 40832917

[15] Getie A, Amlak BT, Ayenew T, Gedfew M (2025) Assessing the impact of telehealth on blood glucose management among patients with diabetes: a systematic review and meta-analysis of randomized controlled trials. BMC Health Serv Res BioMed Cent Ltd 25. 10.1186/s12913-025-12401-9 PMC11840977 39979923

[16] Mannoubi C, Kairy D, Menezes KV, Desroches S, Layani G, Vachon B (2024) The Key Digital Tool Features of Complex Telehealth Interventions Used for Type 2 Diabetes Self-Management and Monitoring With Health Professional Involvement: Scoping Review. JMIR Med Inf 12:e46699. 10.2196/46699 PMC10973964 38477979

[17] Ballesta S, Chillarón JJ, Inglada Y (2023) Telehealth model versus in-person standard care for persons with type 1 diabetes treated with multiple daily injections: an open-label randomized controlled trial. Front Endocrinol (Lausanne) 14. 10.3389/fendo.2023.1176765 PMC10333924 37441496

[18] Lee CS, Tyagi S, Ling Koh EY (2025) Health outcomes of telemonitoring of patients with type-2 diabetes mellitus: One-year results from a randomized controlled trial (Optimizing care of Patients via Telemedicine In Monitoring and aUgmenting their control of diabetes Mellitus). J Telemed Telecare 31:1249–1259. 10.1177/1357633X241261733 39091047

[19] Lee JY, Chan CKY, Chua SS (2020) Telemonitoring and Team-Based Management of Glycemic Control on People with Type 2 Diabetes: a Cluster-Randomized Controlled Trial. J Gen Intern Med 35:87–94. 10.1007/s11606-019-05316-9 31512187 PMC6957608

[20] Guo SHM, Hsing HC, Lin JL, Lee CC (2021) Relationships between mobile ehealth literacy, diabetes self-care, and glycemic outcomes in Taiwanese patients with type 2 diabetes: Cross-sectional study. JMIR Mhealth Uhealth 9. 10.2196/18404 PMC7895642 33544088

[21] Tieu L, Lyles CR, Kim HC (2025) A self-report measure of digital skills needed to use digital health tools among older adults - The Skills Measurement and Readiness Training for Digital Health (SMART Digital Health) Scale. J Am Med Inform Assoc 32:1674–1684. 10.1093/jamia/ocaf151 40971609 PMC12626212

[22] Adu MD, Malabu UH, Malau-Aduli AEO, Malau-Aduli BS (2019) Enablers and barriers to effective diabetes self-management: A multi-national investigation. PLoS ONE 14. 10.1371/journal.pone.0217771 PMC6550406 31166971

[23] Tuobenyiere J, Mensah GP, Korsah KA (2023) Patient perspective on barriers in type 2 diabetes self-management: A qualitative study. Nurs Open 10:7003–7013. 10.1002/nop2.1956 37488987 PMC10495717

[24] Mihevc M, Lukančič MM, Črt Z (2024) Towards Integrated Care for the Elderly: Exploring the Acceptability of Telemonitoring for Hypertension and Type 2 Diabetes Management. Int J Integr Care 24:16. 10.5334/ijic.7621 PMC11100527 38765055

[25] Lee PA, Greenfield G, Pappas Y (2018) Patients’ perception of using telehealth for type 2 diabetes management: A phenomenological study. BMC Health Serv Res 18. 10.1186/s12913-018-3353-x PMC6045870 30005696

[26] German J, Eldridge MR, Esteve L (2025) Perceptions of a comprehensive telehealth intervention in patients with persistently poor type 2 diabetes control. J Clin Transl Sci 9:e153. 10.1017/cts.2025.10082 40895447 PMC12392345

[27] Sun CA, Shenk Z, Renda S (2023) Experiences and Perceptions of Telehealth Visits in Diabetes Care During and After the COVID-19 Pandemic Among Adults With Type 2 Diabetes and Their Providers: Qualitative Study. JMIR Diabetes 8:e44283. 10.2196/44283 37463021 PMC10394605

[28] Gonçalves RL, Pagano AS, Reis ZSN (2023) Usability of Telehealth Systems for Noncommunicable Diseases in Primary Care From the COVID-19 Pandemic Onward: Systematic Review. J Med Internet Res JMIR Publications Inc 25. 10.2196/44209 PMC10022651 36787223

[29] Coningsby I, Ainsworth B, Dack C (2022) A qualitative study exploring the barriers to attending structured education programmes among adults with type 2 diabetes. BMC Health Serv Res 22. 10.1186/s12913-022-07980-w PMC9059690 35501809

[30] Altabtabaei R, Alhuwail D (2023) Exploring the Challenges and Opportunities of Adopting and Using Telemedicine for Diabetes Care and Management: Qualitative Semistructured Interview Study Among Health Care Providers and Patients With Diabetes. JMIR Hum Factors 10. 10.2196/46324 PMC10514770 37676711

